# The Role of EFL Teachers’ Praise and Love in Preventing Students’ Hopelessness

**DOI:** 10.3389/fpsyg.2021.800798

**Published:** 2021-12-21

**Authors:** Fengzhen Liu

**Affiliations:** School of Foreign Languages, Huanghuai University, Zhumadian, China

**Keywords:** teacher praise, loving pedagogy, hopelessness, love, EFL teacher, positive psychology

## Abstract

The emotional dimension of language teaching and learning has recently gained momentum among researchers after pioneering works in positive psychology. Now, teachers’ and students’ emotions play an important role in learning process. Despite the growing body of research on many psychological constructs in L2 education, the role of teacher praise and love in precluding students’ sense of hopelessness about their future and efforts has been largely ignored. Addressing such problems, the present study aimed to examine the definitions, conceptualizations, influencing factors, causes, and outcomes of these three psychological variables in EFL contexts. Moreover, to position the study, this article took a quick glance at the affective trend in education referring to positive outcomes of a loving pedagogy. Finally, different practical implications, research gaps, and future lines of research were provided for passionate researchers.

## Introduction

It is now a widely admitted belief among scholars and practitioners that second/foreign language teaching and learning are both affected by a range of personal emotions, affects, and inner states (Dewaele and Li, 2020; Sikma, 2021). With their important roles in academic contexts, emotions aid in moving one’s academic performance forward, shaping a successful conduct in the classroom, and causing numerous positive academic outcomes (Mercer, 2020). This transition of focus in education and psychology of learning was initiated by two novel trends known as *Positive Psychology* (PP) and its offshoot *Positive Peace Psychology* (PPP). PP capitalizes on the power and value of positive emotions, instead of dwelling on negative stressors, in helping one thrive, flourish, and live a better life (Seligman, 2018; Zhang and Zhang, 2020). It does not linger on negativities and challenges of education but the effective role of positive emotions like joy, engagement, resilience, optimism, hope, passion, care, and the like. On the other hand, PPP as an extension of PP stresses the criticality and impact of positive interpersonal relationships, peace, and harmonious rapport in the class on learners’ academic achievements (Gibson, 2011; Gregersen and MacIntyre, 2021).

It is essential to note that numerous positive academic outcomes develop in a positive and caring educational culture and classroom climate in which the teacher appreciates his/her students’ feelings and individualities and has an effective interpersonal interaction with them (Strachan, 2020; Wang et al., 2021; Xie and Derakhshan, 2021). By this sense of immediacy, teachers cultivate in their learners emotional stability, intrinsic motivation, confidence, resilience, engagement, enthusiasm, enjoyment, and improved performance (Frisby, 2019; Delos Reyes and Torio, 2020; Fathi et al., 2020; Greenier et al., 2021; Han and Wang, 2021; Li and Yang, 2021; Pishghadam et al., 2021; Sun, 2021, among others). These are not solely obtained by caring for learners’ emotions but the actual manifestation of such care in teachers’ classroom behaviors and practices (Derakhshan et al., 2019). One of such indicators is teacher’s positive performance feedback or *praise* which is a verbal sentence, gesture, and an indication of approval that is given after a learner’s behavior involving positive feedback on the desired behavior (Hester et al., 2009). Praise is a free strategy to manage the class, reinforce academic behaviors, and improve engagement, motivation, self-esteem, self-efficacy, and success among the students (Burnett, 2002; Embry and Biglan, 2008; Reinke et al., 2013; Caldarella et al., 2020; Haydon et al., 2020). It is a type of reward which can be used to urge a desired behavior and motivate the learners through an explicit feedback (Firdaus, 2015). Despite its seemingly simple nature, praise requires teachers to have sufficient knowledge and information about its timing, quality, typology, rate of use, and manner of presentation. Otherwise, it changes into a reprimand and a negative factor that increases coercive and disruptive behaviors in the class and causes educational problems that impede learning (Floress et al., 2018).

Nevertheless, a judicial use of teacher praise in the class establishes a democratic atmosphere that helps in creating a pedagogy of love which has long been ignored in language education. Love pertains to the care, appreciation, sensitivity, value, and empathy that an instructor forms in tune with his/her students’ needs, experiences, and academic progress (Yin et al., 2019). It demonstrates itself *via* a caring milieu, harmonious rapport between the teacher and learners, and various classroom practices (Zhao and Li, 2021). The concept had been ignored in educational research due to ethical and professional sensitivities in many cultures until the recent years during which the first stones of this domain were laid by some scholars (e.g., Loreman, 2011; Xie and Derakhshan, 2021). The by-product of this shift was a raise in implementing a loving pedagogy and conducting empirical studies on the concept of love whose results verified the positive impacts of love on L2 students’ motivation, autonomy, engagement, self-esteem, self-efficacy, agency, achievement, criticality, and positive interpersonal skills (Dowling, 2014; Wang and Guan, 2020; Wilkinson and Kaukko, 2020; Fathi et al., 2021; Grimmer, 2021; Xie and Derakhshan, 2021). However, the role of love and praise in preventing and reducing EFL students’ negative emotions has remained an uncharted territory in language education literature. One such emotion is *hopelessness*, which refers to a sense of depression, disappointment, pessimism, and negative expectations about the future (Yenilmez, 2010). It causes both mental and health problems in case it is not eradicated by the teacher and authorities. The root of this destructive factor can be related to family, economy, schools, and teachers. In a classroom culture, which is full of humiliation, reprimand, and mockery, EFL students may find making academic efforts useless due to the toxic environment where they are learning (Wang and Guan, 2020). This may end in quitting education, psychological diseases, and even arousing hatred among the students (Wang and Guan, 2020). Therefore, there is an urgent need to improve teachers’ use and application of praise and love in education, in general, and L2 learning, in particular. Trying to enrich the main literature in this domain, the present article aimed to review the definitions, conceptualizations, benefits, gaps, and practical implications of this line of research in EFL contexts.

## Background

### The Concept of Praise

The notion of “praise” is taken from a Latin word “*pretiare*” which denotes to value/appreciate highly (Shepell, 2000). It includes commending the worth of a behavior/performance through positive feedbacks (Burnett, 2002). As suggested in Firdaus (2015), praise can be seen as an extrinsic reward (verbal or written) that helps control students’ behaviors, performances, and transfer instructional information better. The concept extends beyond “teacher feedback” in that it includes elements of affect, passion, explicit positive performance feedback, and builds on students’ self-image and self-esteem (Zoder-Martell et al., 2019; Haydon et al., 2020). Moreover, as pinpointed by Ennis et al. (2019), praise is a simple strategy to inspire prosocial behaviors among the students and prevent their classroom disruptive behaviors. To put it differently, praise refers to verbal signs of approval succeeding a student’s successful/positive behavior that steps beyond a simple acknowledgment of a correct response (Caldarella et al., 2020). It can also be conceptualized as a reinforce of students’ behavior, an opportunity to boost their motivation, engagement, efficacy, and achievement together with preventing disruptive and problematic behaviors in the classroom (Embry and Biglan, 2008; Reinke et al., 2013; Weeden et al., 2016; Ennis et al., 2019). It is believed that the effectiveness of praise in the classroom largely depends on students’ behavior, teachers’ knowledge of “how” and “when” to use it, classroom rapport, and finally students’ reception of praise as a reinforcer of positive academic performance.

### Types of Teacher Praise

There have been proposed two types of teacher praise in the available literature in this research strand including ***general praise*** (GP) and ***behavioral-specific praise*** (BSP). GP refers to a praise statement or gesture in which the instructor does not determine the desired behavior that aroused his/her praise (Floress and Jenkins, 2015). For instance, saying “good job” “well done,” “perfect,” and “excellent!” or showing a thumbs-up sign after a desired behavior is samples of GP in the classroom. On the other hand, BSP pertains to a statement by which the teacher clearly identifies, names, and specifies the desired behavior that causes the praise by the teacher (Musti-Rao and Haydon, 2011; Zoder-Martell et al., 2019). To exemplify, using sentences like: “I really like the way you…” (e.g., take notes, listen, sit, cooperate, participate, summarize the lesson, and so forth) are all indicative of BSP delivered by the teacher as he/she explicitly mentions the student behavior that generates the praise. As research shows, BSP is more powerful than GP in that it is clearer and more meaningful for the students to continue or change a specific behavior (Brophy, 1981; Zoder-Martell et al., 2019). According to Soto (2014), BSP can be further divided into two sub-types namely, “*praise for effort*” (PFE) and “*praise for ability*” (PFA). PFE refers to teachers’ acknowledgment and admiration of students’ attempts to behave and perform their best in something. It focuses on students’ current process of working and hence improves their work engagement (Chappuis, 2009). For example, saying “you are really working hard on syntax” is a sample of PFE. On the contrary, PFA encompasses teachers’ positive approval of a students’ ability in doing a specific task or what he/she is doing properly. As a case in point, when the teacher admires a student saying “you are really fantastic at writing critical comments on research articles” represents PFA in academia.

Additionally, it is prominent to note that teacher praise can be offered on-task or off-task, meaning it can be delivered for a specific academic student behavior or even disruptive behaviors that cause problems in the class. The more the teachers use BSP, the more the students show on-task behaviors wished through intervention, and the less their disruptive behaviors (Allday et al., 2012; Floress and Jenkins, 2015; Caldarella et al., 2020).

### The Opportunities and Challenges of Teacher Praise

Teachers’ praise in the classroom can offer numerous positive outcomes for the students including an improved classroom participation, engagement, attention, eagerness to learn, and interpersonal relations inside the class. It can be given planned or unplanned yet research reveals that unplanned praise booms students’ motivation and achievement (Brophy, 1981; Soto, 2014). Another strategy to promote the effectiveness of praise is using a combination of verbal and non-verbal (gesture) praise by the teacher instead of a single way of expressing admiration. In addition, as stated in Bandura’s (1977) *Social Learning Theory*, teacher praise can help developing “modeling” practices in the students in that they may model their peer, who has been delivered a praise due to a successful behavior/performance. In a similar manner, teacher praise has the potentiality to generate and enhance many intrapersonal, interpersonal, and psychological factors, such as self-efficacy, self-esteem, identity, enthusiasm, immediacy, self-confidence, and the like.

However, too much delivery of praise by a teacher may does more damage than good in the sense that it promotes teacher dependency, extrinsic motivation orientation, and controlled practice in the learners (Saeverot, 2011; Soto, 2014). Likewise, reliance on teacher’s praise can decline and even deter students’ autonomy, agency, self-regulation, intrinsic motivation, self-gratification, self-directed learning, and self-improvement as they get used to making attempts to satisfy their teachers’ expectations and desires without discovering the joy by themselves (Chappuis, 2009; Saeverot, 2011).

### Critical Factors Influencing Teacher Praise Effectiveness

**Figure 1:**
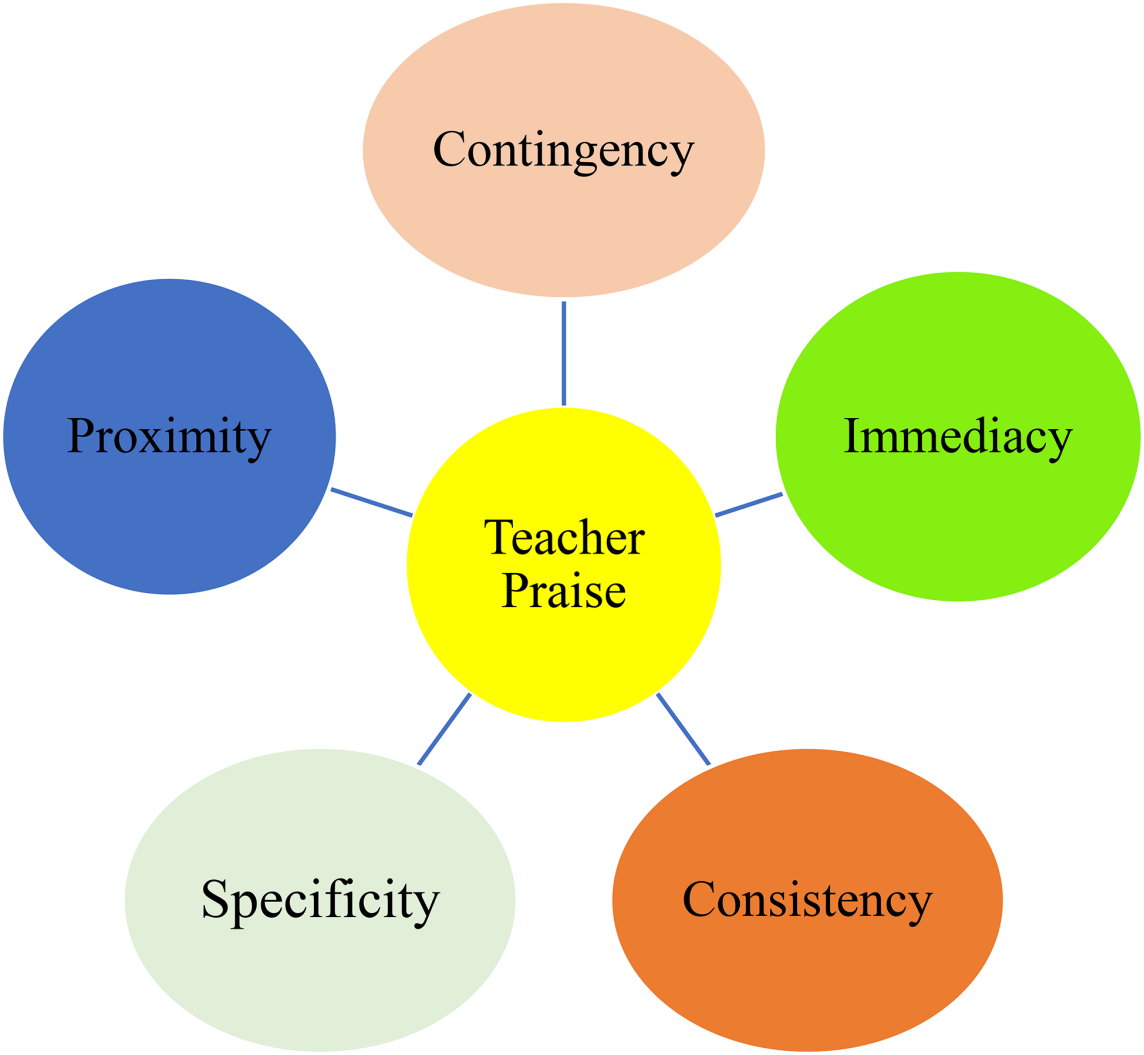
Factors influencing teacher praise effectiveness.

The construct of praise is by no means a simple strategy to control or manipulate the learners but a research-based, naturalistic technique to develop positive and meaningful interpersonal communication and rapport between the teacher and his/he students in the class (Haydon et al., 2020). Aside from the typologies of teacher praise, educators and practitioners must take into consideration a number of critical factors that significantly affect the quality and efficacy of their given praise to the students. They include *contingency*, *immediacy*, *proximity*, *consistency*, and *specificity* (Figure 1). Contingency argues that teacher praise is best offered when it is connected and contingent upon students’ target behavior (Cooper and Scott, 2017). Moreover, immediacy concerns the timing of providing the praise suggesting that it is best to deliver the praise immediately after the desired behavior occurs without any delay (Hattie and Timperley, 2007). Another important factor in praising is proximity, which refers to the physical distance between the teacher and students suggesting that a close proximity is most suitable for teacher praise in that it blocks attentional distractions among the students. Furthermore, teacher praise must have consistency in the sense that a praiseworthy behavior of a student must be delivered predictable, constant, and systematic admiration of the teacher; otherwise, it makes students perplexed whether their performance/behavior is appropriate or not anytime they act (Alberto and Troutman, 2009). The final critical factor that extremely influences the effectiveness of a teacher’s praise is specificity, which suggests that teachers’ praise delivery must be task/behavior specific in the sense that informative feedbacks should be given to a pre-specified, desired behavior/performance so that students improve in that specific area of knowledge/skill (Haydon et al., 2020).

### The Definition of Love

Defining the concept of love is not as easy as it appears. It is a complex word, which means different things across different contexts and cultures. Although it is an abstract variable, it is full of meaning, emotion, affection, and depth (Grimmer, 2021). Most people have diverse understandings and descriptions for love limiting its application to only the private and familial realm (Vincent, 2016). In education, talking about love has traditionally been viewed as “stepping beyond the boundaries” linking it sexual desire (Cousins, 2017). This understanding is now replaced by “love as both an emotion and an action”, which paved the way for the later introduction to professional love. Aside from the private sphere of family, love can extend to other areas, such as job, foods, friends, nature, and so on (Loreman, 2011; Smith, 2011). Talking about professional love is still a debatable issue in many cultures drawing on the old belief that one needs to draw a demarcation line between his/her personal inner emotions and the professional milieu where he/she works (Smith, 2011). This claim runs counter to the nature of human as a social creature since it is implying a separation between personal self and social-professional self. Now, the concept of love has got an identity for itself in the lexicon of education referring to teachers’ pure kindness, affection, empathy, and care toward their students’ emotions, learning, and development (Maatta and Uusiautti, 2012; Zhao and Li, 2021). It can improve students’ academic achievement and performance as long as it strictly follows ethical and professional etiquettes.

### Conceptualizations of Love and Affect in Pedagogy and Education

The root of considering and investigating students’ and teachers’ emotional states/factors in education belongs to a new trend in psychology known as *positive psychology* (PP) that foregrounds the way people can thrive and live happier (MacIntyre et al., 2019; Wang et al., 2021). In contrast to traditional trends in general psychology which solely maneuver over negative emotions, PP encourages the practitioners to focus on the potentialities of positive emotions (MacIntyre and Mercer, 2014; Pishghadam et al., 2021). This movement itself has been motivated by another trend named *affective pedagogy* (AP), which is a manner of teaching intended to provoke specific emotional states (Ainsworth and Bell, 2020). AP grew out of a broader paradigm in education named *the affective turn* (Clough, 2007) that underscores the criticality of affective experiences and states in the process of learning. AP capitalizes on psycho-pedagogy among the teachers to enhance their students’ inner feelings/emotions, which are necessary for their academic success (Williamson, 2016). Both schools substantiated the linkage of emotions and learning success in which the teacher and students have intimacy and a positive, caring rapport with each other (Patience, 2008).

An offshoot of such a democratic and friendly relationship in the class is the establishment of pedagogical love that has an important role to play in improving students’ emotions, social competency, personality, and psychological wellbeing (Yin et al., 2019) as well as other positive psychological components, such as self-compassion and job wellbeing (Rajabi and Ghezelsefloo, 2020). First and foremost, a point worth mentioning is that the construct of “love” can find its way to the traditional Chinese education philosophy prosed by Mencious, a Chinese ancient Saint who once argued that “the benevolent loves others” (Ri Zhe Ai Ren in Chinese, Feng, 2012). That is to say, those people with perfect virtue have universal love. In alignment with this, the diverse conceptualizations of “love” have been given since 16th century (Zhao and Li, 2021). As eloquently put by Loreman (2011), love is a complicated variable that can be regarded as a rigorous learning motivator, a sign of effective education, the heart of classroom communications, and an inherent human need. Moreover, owing to its association to many aspects in human life, the notion has been described and explicated *via* different psychological, religious, and philosophical lenses. As a case in point, Sternberg (1986) proposed a tripartite theory for the construct of love including three elements of *intimacy*, *passion*, and *decision/commitment*. According to him, a sound and effective kinship in education, therefore, are one wherein these three elements are united. Taking a different stance, religious perspectives generally consider love as divine and a property of God (Zhao and Li, 2021). Finally, philosophical conceptualizations proposed by Plato and Aristotle clarified love as a quest for beauty that can be of three types: (a) *eros* (i.e., sexual love), (b) *philia* (i.e., love of friends), or (c) *agape* (i.e., love of mankind).

**Figure 2:**
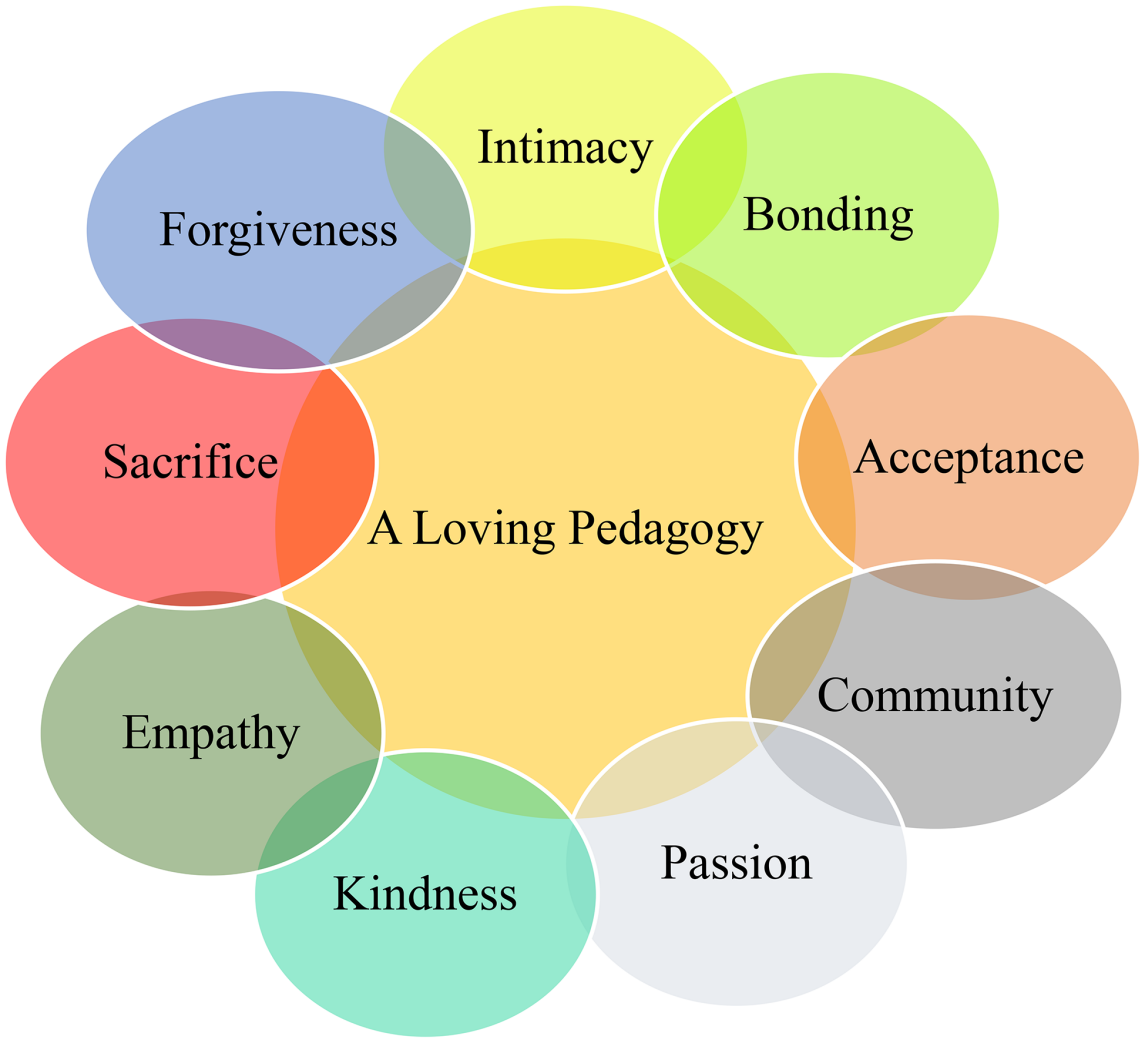
Nine concepts constituting a loving pedagogy.

Despite its socio-cultural and historical origins, the concept of love has long been a taboo or sensitive term in education until some pioneering studies broke the ice and overstepped the strictly unapproachable bond between love and education (e.g., Loreman, 2011; Akkaraju et al., 2019; Wilkinson and Kaukko, 2020). This led to the coinage of a new concept known as “loving pedagogy” (Xie and Derakhshan, 2021). In his seminal work on loving pedagogy, Loreman (2011) considered love as momentous for educators and proposed nine concepts related to love comprising *passion*, *kindness*, *empathy*, *intimacy*, *bonding*, *sacrifice*, *forgiveness*, *acceptance*, and *community* (Figure 2). In an educational context that is oriented toward students’ emotions, needs, and expectations, a democratic and caring relationship are established which, in turn, boosts other aspects of teaching and learning. In sum, a pedagogy of love combines the mentioned nine concepts and is an external force that impacts individuals’ spirit, social and emotional states, academic achievement, engagement, and interpersonal communication skills (Loreman, 2011). At the same time, a love-based instruction resolves many educational problems and pitfalls, such as stress, tension, boredom, hopelessness, anxiety, and shyness to name a few. One of the venues for loving pedagogy to occur is frequently but logically using praise in the class to constitute a caring environment where students feel safe and relaxed to take academic risks and initiatives.

### Hopelessness: The Definition, Causes, and Outcomes

In the available literature on the variable of hopelessness, limited definitions have been offered by scholars owing to its scanty exploration. Nevertheless, the concept refers to a negative feeling and anticipation about one’s future which takes roots form his/her negative attributional styles and experiences (Rice et al., 2006; Yenilmez, 2010). In academic contexts, it forms a sense of pessimism in the students about their future and this, in turn, affects their degree of motivation, interest, and effort (Pekrun et al., 2009). As for the causes of this negative stressor, research points to a number of causative factors, such as the very person him/herself, his/her family, socio-economic status, the teacher, the school climate, the academic staff, peers, and finally the materials used in education. All these parties play a crucial role in shaping and eradicating the sense of hopelessness in a student. It is widely admitted that a student’s academic success depends on various personal and contextual factors, which can degenerate his/her motivation for learning and blur their future outlook.

If not properly and practically dealt with in academia, this devastating feeling can culminate in dire outcomes, such as depression, anxiety, stress, tension, isolation, social separation, boredom, shyness, shame, identity crisis, anger, and even suicide (Rice et al., 2006; Taskesen et al., 2012; Ismail, 2015; Lew et al., 2019). On the contrary, hopelessness has a negative association with motivation, engagement, self-efficacy, self-esteem, wellbeing, academic success, enjoyment, and perfectionism in education. As expected, many positive outcomes in education, in general, and language learning, in particular, depend on the absence of this damaging construct in learner psychology. This calls all educational systems worldwide to form a loving atmosphere and take suitable actions against the construction of negative factors in students for whom the education is carried out. Otherwise, all the attempts will work in vain.

### Implications, Research Gaps, and Future Directions

This review article can have many practical implications for different parties including EFL teachers, students, teacher educators, policy-makers, and researchers interested in learner/teacher psychology. The propositions made in this article are momentous for EFL teachers in that they can enhance their awareness, understanding, and use of praise and a pedagogy of love in the context of language education and eradicate negative factors like hopelessness in learners. By identifying the criticality of praise and love in education, EFL teachers can employ appropriate techniques to fight against students’ hopelessness and reduce its degree (Wang and Guan, 2020; Han and Wang, 2021). Likewise, EFL students can benefit from the ideas of this study in that they can understand the value and importance of their emotions in language education, which is by no means an emotion-free occupation these days. They can assist their teachers in forming a friendly, democratic, and love-based educational atmosphere in which the students can wipe out negative emotions, devise new life scripts, and attain positive outcomes. Teacher trainers can run workshops and professional development courses for novice EFL teachers where beneficial strategies and techniques about how to cope with students’ negative and positive emotions and the impact of teacher’s praise and loving pedagogy are taught carefully. Other than pedagogical issues, teachers can learn about many important psychological and emotional factors in such training programs.

At the macro level, policy-makers and those in charge of planning for education can revisit their understanding of love, especially its application in education and help academic staff and practitioners in shaping a pedagogy of love in which students’ emotions and experiences are really cared about instead of being seen as emotionless creatures who must only listen and do as their teacher says. Finally, language researchers can use this study as a starting point in running comparable studies on this line of research in EFL/ESL contexts across cultures and religions. As stated in the literature, most of the studies in this domain are in general education context and few studies (if any), in L2 context, have explored the preventive role of teacher praise and love in blocking and reducing students’ sense of hopelessness. Hence, future research can be done on this strand using questionnaires, interviews, and classroom observations to provide a triangulated data on the issue. Since love and praise may have different conceptualizations in different cultures, cross-cultural studies can also be conducted using diaries and life stories. Likewise, longitudinal research is suggested to avid scholars in this area to unpack the developmental process of hopelessness, its causes, correlates, and solutions. Additionally, correlational studies are rare in EFL contexts regarding teacher praise and love along with many other positive psychologies driven emotions like enjoyment, passion, resilience, credibility, immediacy, stroke, care, pride, optimism, resilience, and the like. Future researchers can fill the existing gaps and instead of running one-shot studies, take advantage of qualitative designs to provide a deeper insight about praise, love, hopelessness, and other negative factors like boredom, shyness, shame, and fear. All these backdrops indicate that this area of inquiry is still fertile for cultivating research in EFL contexts.

## Data Availability Statement

The original contributions presented in the study are included in the article/supplementary material, and further inquiries can be directed to the corresponding author.

## Author Contributions

The author confirms being the sole contributor of this work and has approved it for publication.

## Conflict of Interest

The author declares that the research was conducted in the absence of any commercial or financial relationships that could be construed as a potential conflict of interest.

## Publisher’s Note

All claims expressed in this article are solely those of the authors and do not necessarily represent those of their affiliated organizations, or those of the publisher, the editors and the reviewers. Any product that may be evaluated in this article, or claim that may be made by its manufacturer, is not guaranteed or endorsed by the publisher.
